# P44 Nosocomial neurosurgical meningitis due to XDR *Pseudomonas aeruginosa*: clinical experience with cefiderocol

**DOI:** 10.1093/jacamr/dlac004.043

**Published:** 2022-02-16

**Authors:** D. R. Stevenson, B. P. Cherian, M. Kinzig, F. Sörgel, D. W. Wareham

**Affiliations:** Royal London Hospital, Barts Health NHS Trust, London, UK; Royal London Hospital, Barts Health NHS Trust, London, UK; IBMP, Institute for Biomedical and Pharmaceutical Research, Nürnberg-Heroldsberg, Germany; IBMP, Institute for Biomedical and Pharmaceutical Research, Nürnberg-Heroldsberg, Germany; Royal London Hospital, Barts Health NHS Trust, London, UK; Queen Mary University of London, UK

## Abstract

**Background:**

Carbapenem resistance due to MBL production is relatively uncommon in *Pseudomonas aeruginosa* infections in the UK. We report a case of post-operative meningitis caused by a New-Delhi MBL-producing (NDM-1) strain, outlining the microbiological and management challenges.

**Patient case:**

The patient was admitted to intensive care after craniotomy and evacuation for an intracerebral haemorrhage. Three weeks later she was febrile due to nosocomial meningitis. Purulent material evacuated from extradural and subdural spaces cultured *P. aeruginosa*, along with blood cultures and CSF. Susceptibility testing as per EUCAST methodology indicated the isolate was resistant to all β-lactams, aminoglycosides and quinolones but susceptible to colistin. Susceptibility to cefiderocol by disc diffusion was in the area of technical uncertainty at 21 mm (S≥22 mm). Immunochromatographic flow testing (CARBA5 NG Biotech) detected an NDM carbapenemase. WGS (Illumina) confirmed the presence of *bla*_NDM-1_ and a plethora of AMR genes responsible for the XDR phenotype {cephalosporins (*bla*_PDC-10_, *bla*_OXA-488_), aminoglycosides [*aph(3')-VIa*], quinolones (*crpP*), macrolides [*mph*(E), *msr*(E)], phenicols (*catB7*) and fosfomycin (*fosA*)}. No mutations in PBPs associated with resistance to β-lactams or iron acquisition systems involved in uptake of siderophore conjugated antibiotics were identified. The isolate belonged to ST1047, serotype 07, previously identified as a high-risk epidemic clone originating in Myanmar.

**Results:**

Treatment was commenced with IV and intrathecal colistin as well as IV cefiderocol (Figure 1); a novel siderophore cephalosporin licensed for Gram-negative infections with limited treatment options. No synergy between the two drugs could be demonstrated in chequerboard assays. Although CSF sterility was achieved on three subsequent cultures with clinical response, the patient sadly died due to the underlying intracerebral injury. Four other patients subsequently became colonized with this strain and infection control measures (twice weekly screening of all patients, environmental cleaning, water testing, isolation of cases) were instituted, bringing the outbreak under control. Variable number tandem repeat analysis linked these strains to that of another patient discharged from the unit 9 months prior, but not previously reported in the UK.

**Conclusions:**

The first problem encountered was a lack of evidence for the CNS penetration of cefiderocol and efficacy in the treatment of meningitis. The external ventricular drain required to administer intrathecal colistin enabled us to monitor CSF cefiderocol levels. Five CSF samples were shipped at −80°C for analysis in Germany by mass spectrometry. CSF cefiderocol levels of 1.22 μg/mL, 1.34 μg/mL, 2.39 μg/mL, 3.53 μg/mL and 3.90 μg/mL were disappointingly close to the pharmacodynamic breakpoint of 2 μg/mL and only available after treatment withdrawal. Dosing of cefiderocol was initially cautious starting at 1 g/8 h due to a creatinine clearance of 53  mL/min but later increased to 1.5 g/8 h. Further dose increases guided by serum and CSF monitoring could feasibly lead to therapeutic CSF cefiderocol levels. Clinical and pharmacological studies are needed in this area. To our knowledge this is the first attempt to treat a case of XDR neurosurgical meningitis using cefiderocol with precision treatment and achieving microbiological cure.

**Figure 1.**
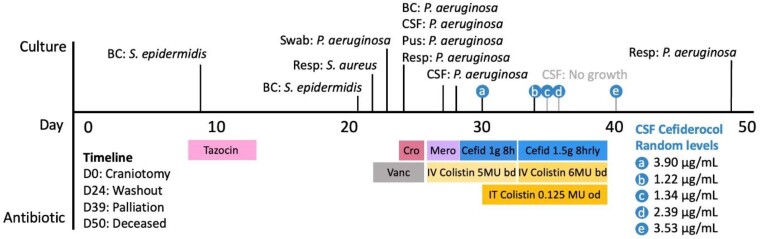
Clinical timeline, culture results, antimicrobial regimens and cefiderocol levels. BC, blood culture; Resp, respiratory specimen; Swab, craniotomy swab; Pus, subdural and extradural pus; Cro, ceftriaxone; Mero, meropenem; Cefid, cefiderocol; Vanc, vancomycin; IT, intrathecal; MU, million units; bd, two times a day; od, once daily.

